# Artificial intelligence-assisted vocabulary teaching: a meta-analysis study

**DOI:** 10.3389/frai.2026.1806700

**Published:** 2026-04-23

**Authors:** Bilal Şimşek, Bekir Direkci, Betül Koparan, Serdar Akbulut, Emine Ela Şimşek, Mevlüt Gülmez, Damla Nur Ökmen

**Affiliations:** 1 Department of Turkish Language Education, Faculty of Education, Akdeniz University, Antalya, Türkiye; 2 Department of Turkish Language Education, Faculty of Education, Manisa Celal Bayar University, Manisa, Türkiye; 3 Department of Early Childhood Education, Faculty of Education, Akdeniz University, Antalya, Türkiye

**Keywords:** artificial intelligence, language learning, meta-analysis, the use of technology in language learning, vocabulary acquisition

## Abstract

**Introduction:**

The literature reports promising findings on the use of artificial intelligence (AI) to enhance vocabulary learning performance. However, this body of research is still relatively recent, important gaps remain, and findings vary across studies. Therefore, synthesizing the available evidence is essential to reach more generalizable conclusions and to gain deeper insight into AI-supported vocabulary instruction.

**Methods:**

This study employed a meta-analytic design to examine the effects of AI-supported interventions on individuals’ vocabulary learning performance. Articles published between 2015 and 2025 were retrieved from the Web of Science Core Collection (WOS), Scopus, and ERIC databases. Based on the inclusion criteria, 18 studies yielding 23 effect sizes and involving a total of 1,823 participants were included in the analysis.

**Results:**

The findings showed that AI-supported interventions had a positive and large overall effect on vocabulary learning performance (*g* = 1.309). This result indicates that AI applications significantly improve individuals’ vocabulary learning outcomes. Moderator analyses revealed significant differences according to language type, learner educational level, control treatment, and intervention setting. In contrast, teacher involvement level and intervention duration did not produce statistically significant moderating effects.

**Discussion:**

Overall, the results suggest that AI-supported vocabulary instruction is highly effective in improving vocabulary learning performance. These findings indicate that AI applications can provide context-sensitive and personalized learning opportunities across different educational settings. The study contributes to the growing literature by offering a comprehensive synthesis of current evidence and highlighting the instructional potential of AI in vocabulary teaching.

## 1 Introduction

In recent years, the increasing trend of ‘artificial intelligence’ has rapidly increased in use and prevalence in all areas of life, including education and particularly in challenging fields such as language education. Vocabulary teaching, one of the most difficult aspects of language teaching, has also become part of this trend, and artificial intelligence (AI) supported vocabulary teaching studies and applications have become widespread. Meta-analyses examining scientific research on this subject also emphasise the positive effects of AI on language learning and its significant contribution to vocabulary learning in particular (Torres and Kahveci, 2025: *g* = 0.74; Xu and Wang, 2024: *g* = 0.812).

In language education using AI tools; positive effects on language proficiency, motivation, and participation (Kasap, 2024: 0.58, 0.44, 0.44); a significant improvement in academic achievement at the K-12 level (Temiz and Kafadar, 2025); and increases in fluent (accurate) communication and vocabulary learning (Wang, 2025). However, different meta-analysis studies show that the effect sizes of AI-support vary significantly depending on the context (Torres and Kahveci, 2025; Xu and Wang, 2024), and individual studies also show that this variation spans a wide range (moderate to large). Particularly regarding vocabulary acquisition, different results emerge. While a 40% improvement was observed in a study using AI-supported vocabulary teaching (Wang, 2025), another study found that the use of AI-supported flashcards lagged behind traditional flashcard methods, especially in retention tests (Temiz and Kafadar, 2025). Although AI has been seen to support language skills, there are also difficulties arising from limited contextual information, particularly in vocabulary usage. This situation demonstrates that in language learning, it is not sufficient to merely recognise or remember words; it is also important to use them in the correct contexts (Wiboolyasarin et al., 2025). One reason for the differences observed between studies is the degree to which the tools are customised and personalised for this purpose. Personalised applications based on RNN and NLP showed positive increases in language proficiency (Ma et al., 2025); increases in vocabulary recall and syntactic accuracy (25 and 30%, respectively) (Aravind et al., 2025), while non-customised e-learning methods are seen as less effective and engaging (Yang et al., 2025). The real-time feedback and dynamic content adjustment (difficulty level, interaction, or engagement, etc.) offered by these personalised systems stand out as factors behind the increased performance (Sripada et al., 2025; Feng, 2025). It is necessary to better understand these observed differences and how the differences in research processes affect the AI-supported vocabulary learning process (Kasap, 2024; Temiz and Kafadar, 2025).

The conflicting findings in the current literature can be seen as a reflection of the methodological and conceptual limitations of the studies. Systematic reviews have found that randomised controlled trials are generally not applied (Qiao et al., 2025) and that AI-supported studies, despite their positive outcomes, do not appear to be free from bias (Paglialunga and Melogno, 2025). These methodological limitations do not allow for generalisations that could reveal whether the positive effects seen in the studies are due to natural development or the effect of AI. Upon examination, it was found that studies were conducted with homogeneous and small groups over short periods (Agnes and Srinivasan, 2024) or as cross-sectional studies (Ortega-Ochoa et al., 2024), and the majority of studies were conducted with weak experimental designs without a control group (Qiao et al., 2025). Furthermore, another limitation for generalisation is that the measurements in the studies are generally based on participants’ subjective perceptions, satisfaction, and attitudes, with objective performance assessments being in the minority (Karri et al., 2025). Alongside these methodological limitations, the linguistic proficiency of participants (Torres and Kahveci, 2025), the degree of personalisation of AI tools (Ortega-Ochoa et al., 2024), and the L1-L2 language effect may also contribute to this variation.

Fully understanding the impact of AI in vocabulary teaching requires a meta-analysis study to resolve the contradictions and uncertainties present in the literature. Examples of meta-analyses in the field of technology-supported vocabulary learning (Lin and Lin, 2019; Şimşek and Şimşek, 2025; Yu and Trainin, 2022) have successfully explained the effect sizes and the impact of moderators in individual studies. Meta-analyses, using random effects models, help to view the effects of methodological and conceptual variables as a generalisable pattern (Ekizer, 2025; Zhou and Zhou, 2026). In this context, the present study conducted a comprehensive meta-analysis encompassing a random effects model, subgroup analyses, heterogeneity assessment, and publication bias tests (Borenstein et al., 2009) to aim to produce a reliable effect size estimate regarding the effectiveness of AI-supported vocabulary teaching, identify the main factors shaping this effect (student profile, type and duration of intervention, linguistic context, degree of personalisation, etc.), and assess whether there is a potential confirmation bias in the literature.

## 2 Literature review

As inputs increase, learning information becomes easier. Therefore, the Dual Coding Theory (DCT), which explains this system, forms the basis of AI-supported vocabulary learning, where both verbal and visual inputs come into play and multiple cognitive channels are active (Clark and Paivio, 1991; Schnotz et al., 2012). The applications of this theory are evident in effective multimedia learning, where multiple cognitive channels are active, through principles such as coherence, spatial/temporal contiguity, modality, and redundancy (Devers et al., 2018; Schnotz et al., 2012). Essentially, these principles focus on managing learners’ cognitive load by adjusting the harmony between text and visual elements. In digital learning, DCT is realised through the support of visuals with text (Alatawi et al., 2025) and the addition of auditory stimuli alongside visual stimuli (Supitayakul et al., 2023). AI technologies make the DCT theoretical framework visible by ensuring the harmony of visual and auditory modes through digital humans and other characters (Wang and Li, 2025). Another effect of DCT in terms of language teaching is that it supports interlingual transfer and development in terms of word recall/use by adapting to bilingual and multilingual contexts (Paivio, 2014; Kanellopoulou et al., 2019).

The increasing linguistic diversity and the widespread adoption of technology-supported learning approaches in today’s world make the use of multimedia in education inevitable. In this context, adapting multimedia learning principles to learners’ needs and contextual learning environments has become crucial (Kartal, 2010). In an experimental study on word acquisition, Cuevas and Dawson (2018) found that, compared to traditional learning styles (e.g., visual, auditory), the meaningful integration of visual and verbal elements (DCT) yielded more meaningful results. Contrary to this positive reference, DCT is sometimes criticised due to its limitations in abstract word contexts. Paivio (2013) stated that the theory is based on concrete imagery, does not sufficiently support abstract and emotional content and contexts, and has weak explanatory power in this area. Despite these criticisms and limitations, AI-generated visual and auditory materials appear attractive in vocabulary teaching due to their advantages in rapid production, context-specific personalisation, ease of use, and accessibility. However, there are not yet enough studies comparing the effectiveness of AI-generated content with human-made content (Alatawi et al., 2025). This situation demonstrates the critical importance of controlling the quality of multimedia content produced by AI.

The ‘Mobile-Assisted Language Learning’ (MALL) framework is based on the situated learning theory, which posits that knowledge is a product of the context/culture in which it is learned and used (Brown et al., 1989). MALL facilitates vocabulary acquisition by providing an authentic, social, context-sensitive, and personalised learning experience based on mobile technologies (Kukulska-Hulme and Shield, 2008; Lin and Lin, 2019). Context-sensitive AI mobile applications offer an authentic learning environment through methods such as translating the smartphone’s language into the target language (L2, etc.) (Ye and Shi, 2026), location-based content generation (Horst et al., 2025), and vocabulary learning with real-world materials (Lu et al., 2025; Ye et al., 2023). The Mobile-Assisted Seamless Vocabulary Learning (MASVL) framework also systematises the process by integrating continuous and context-sensitive vocabulary learning/acquisition with real life (Bai et al., 2025). However, when gamified language and vocabulary learning mobile applications do not align with the real world (e.g., Duolingo & BaiCiZhan, etc.), context collapse may occur, and the transfer of vocabulary knowledge becomes difficult (Gao and Pan, 2023). Although AI-supported mobile applications attempt to mitigate this with dynamic difficulty and reward adjustments based on individual progress (Kherazi and Bourray, 2024), they lag behind traditional methods in context-sensitive productive vocabulary skills. Nevertheless, these applications are superior to traditional methods in terms of engagement and recall (Alisoy and Sadigzade, 2025). Indeed, MALL strategies dynamically balance these two approaches by supporting vocabulary acquisition through both incidental learning in everyday contexts and intentional learning techniques such as structured activities and spaced repetition. Thus, they aim to increase vocabulary learning success in both the short and long term (Lin and Lin, 2019; Dewi et al., 2020). As a result, the gaps in recall and usage between in-app learning and productive language use in real life are bridged by blended approaches that combine mobile learning with classroom learning (Alisoy and Sadigzade, 2025). Peer support, language switching, and social interactions play a critical role in vocabulary teaching, particularly in multilingual contexts (Hu and Hassan, 2025).

AI-based vocabulary learning applications may enhance learners’ attention to target vocabulary through adaptive and personalised input (Karri et al., 2025). These systems can guide learners’ attention to target vocabulary through various forms of input enhancement. Such features may contribute to vocabulary learning by increasing exposure and supporting deeper processing of lexical items. Liu et al. (2021) state that the effectiveness of these strategies and the extent to which they are utilised show a linear relationship with learners’ working memory capacity, while Kang et al. (2024) have determined that increased font size used in context yields better results than other approaches. In addition, in interactive contexts, AI chatbots support implicit learning by correcting learners’ errors within context through conversational recasts (Wiboolyasarin et al., 2025). Providing real-time feedback and generating natural language usage by AI improves vocabulary and productive language skills (Alshehri, 2025). Furthermore, AI systems enhance vocabulary learning by seamlessly integrating visual and auditory content. Yu (2025) demonstrated that this integration has more meaningful positive effects on vocabulary learning and recall than do contextual and intrinsic strategies.

While recent studies provide valuable insights into AI-supported vocabulary learning, it should be noted that a considerable portion of this literature reflects emerging and rapidly evolving applications of artificial intelligence. As such, some reported findings, including unusually large effect sizes (e.g., *g* > 3), should be interpreted with caution in terms of methodological plausibility and generalizability. These variations may be associated with factors such as small sample sizes, short intervention durations, or context-specific instructional designs. Therefore, there is a clear need for a systematic and comprehensive evaluation of this evolving body of research. In this regard, the present meta-analysis aims to provide a more balanced and generalizable estimate of the effectiveness of AI-supported vocabulary learning by synthesizing findings across studies and examining potential sources of variation.

### 2.1 Current study

This meta-analysis study aims to examine the effect of AI-supported applications on students’ vocabulary learning from a holistic perspective. The research questions were formulated in alignment with the PICOS framework to ensure clarity and systematic structure. In line with this fundamental objective, the study was guided by the following two research questions:

1. What is the overall effectiveness of AI-supported interventions on vocabulary learning?
2. Do the identified moderator variables create a meaningful difference in the effect of AI interventions on vocabulary teaching?

## 3 Methods

In the current meta-analysis, we utilised the Comprehensive Meta-Analysis Software (Version 2.2.064) programme to analyse the data. In the process of systematically collecting, rigorously evaluating, and reporting empirical evidence on the effectiveness of AI-supported interventions in the context of vocabulary teaching, we followed these steps: literature review and study identification, eligibility assessment and exclusion, development of a coding guide, calculation of effect sizes, and moderator analyses. Detailed explanations of these application stages and the methodological flow we followed are presented under the following subheadings.

### 3.1 Search and retrieval of studies

Before commencing the current meta-analysis, we first determined which databases to search. We selected the Web of Science Core Collection (WOS), Scopus, and ERIC databases due to their extensive data pools (Chang et al., 2022), high-quality academic literature (Zhu et al., 2024), and consideration in meta-analysis studies conducted by different researchers (Mohsen et al., 2024; Tsai and Tsai, 2018). We selected the Web of Science Core Collection (WOS), Scopus, and ERIC databases. We then limited the years we would search to 2015–2025. The rapid advancement in AI technology and changes in content were significant factors in determining this limitation. We then identified keywords relevant to the purpose of our meta-analysis to conduct the search within the framework of the databases we selected and the years we limited the search to. To ensure transparency and reproducibility, the literature search strategy was explicitly defined and adapted for each database. The search strings, search fields, and filtering procedures used for Web of Science, Scopus, and ERIC are provided in detail in Appendix A. The entire search process was carried out by two researchers. We compared the data we obtained and created a final pool. We then proceeded to the inclusion and exclusion criteria stage.

### 3.2 Study eligibility: inclusion and exclusion criteria

When determining which studies to include in this meta-analysis, we applied certain selection criteria to ensure the validity and reliability of the results. These criteria are as follows:

1. Journal articles to be included in this study must be searched in the ‘Web of Science Core Collection (WOS), Scopus and ERIC’ electronic databases.
2. Studies must have been published between 2015 and 2025.
3. The studies must directly examine the effect of AI technology on vocabulary teaching.
4. The studies must only have an experimental or quasi-experimental design. Qualitative, descriptive, or studies that present only a theoretical framework are not included in the meta-analysis.
5. The study must have been conducted with clearly defined experimental and control groups. AI-supported vocabulary teaching should be implemented in the experimental group, but no AI-supported intervention should be included in the control group.
6. Participants in the studies should be individuals with normal development, enrolled in any level of education from pre-school to university.
7. All studies prepared for the teaching of different foreign languages should be included in the meta-analysis. These studies may focus on vocabulary teaching in an L1 or L2 context.
8. The statistical data required to perform the meta-analysis should be fully reported in the studies included in the analysis.

To enhance methodological transparency and ensure a systematic structure, the inclusion criteria of this meta-analysis were defined in accordance with the PICOS framework (Population, Intervention, Comparison, Outcomes, and Study Design), which is widely recommended in systematic reviews. The population (P) included learners from preschool to higher education levels, reflecting the broad applicability of AI-supported vocabulary learning across educational contexts. The intervention (I) consisted of AI-supported applications designed to enhance vocabulary learning, while the comparison (C) group included traditional instructional methods as well as technology-supported instructional practices that do not involve artificial intelligence. The outcomes (O) focused on measurable vocabulary learning performance, including test scores and achievement indicators. Finally, the study design (S) was limited to experimental and quasi-experimental studies with control groups to ensure methodological rigor. The use of the PICOS framework allowed for a structured, transparent, and replicable study selection process.

**Figure 1:**
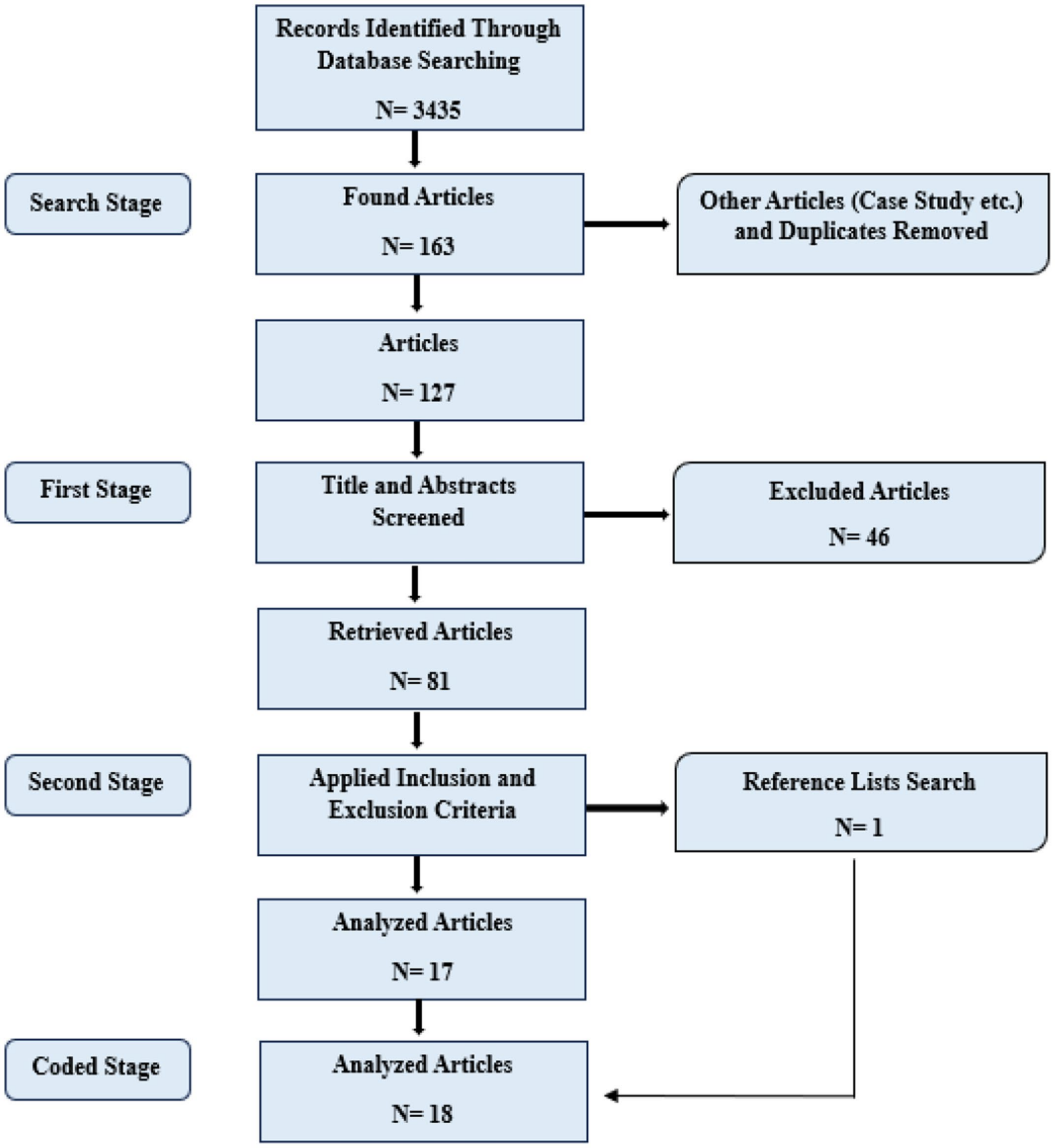
Diagram depicting the literature search and screening procedure.

We followed a systematic workflow to identify the studies included in the meta-analysis. First, we determined the keywords. We then conducted a search in the electronic databases we had identified. In the initial search using the relevant keywords, we selected 163 studies from a total of 3,435 studies. We excluded duplicate studies and studies focused on qualitative data, leaving 127 studies. We then carefully screened the titles and abstracts of these studies. During this process, we excluded 46 publications that did not involve AI-supported intervention and focused on skills other than word teaching, reducing the number to 81. In the final stage, we thoroughly examined the full texts of the remaining studies according to our criteria. At this stage, we excluded 29 studies from the meta-analysis that did not meet our inclusion criteria or lacked sufficient data on experimental results. Additionally, after screening the reference lists of the studies we reviewed, we included one new study that met our criteria in the meta-analysis. As a result, a total of 18 studies were included in the meta-analysis through independent review and consensus among the researchers. To further ensure the reliability of the study selection process, inter-rater agreement between the two researchers was assessed using Cohen’s kappa coefficient. The analysis indicated a high level of agreement (*κ* = 0.92), demonstrating strong consistency in screening decisions. Any discrepancies were subsequently resolved through discussion until consensus was achieved. This meta-analysis was conducted in accordance with the Preferred Reporting Items for Systematic Reviews and Meta-Analyses (PRISMA 2020) guidelines. A detailed PRISMA flow diagram of the literature search and selection procedure is presented in Figure 1.

### 3.3 General coding procedure

We coded the studies included in this meta-analysis under various headings: These headings include: biographical information, keywords, basic study information indicating the research method, potential moderator information, sample sizes, means, standard deviations, *t*-values, *p*-values, and information necessary for calculating effect sizes such as Cohen’s *d*-values. Two researchers performed the coding separately. During the coding process, the researchers met regularly and resolved any differences that arose through discussion. When necessary, they re-examined the studies and ensured the reliability of the coding. The final coding list showed 100% agreement between the researchers.

The moderators considered in this study were as follows: language type, learner educational level, teacher involvement level, control treatment, intervention duration, intervention setting. These moderators were determined by the researchers after reviewing the literature. We first grouped the studies included in the meta-analysis in terms of language type. We found that the vast majority of studies focused on L2. There were a small number of studies focusing on L1. Furthermore, all studies focusing on L2 focused on English vocabulary teaching. Studies in the L1 context focused on Korean vocabulary teaching. Therefore, there were no additional studies focusing on a different language that met our criteria. The number of studies focusing on vocabulary teaching in the L1 and Korean contexts within these groups was very small, but we still wanted to see the difference and suggested that the results should be carefully evaluated. We coded the learner educational level moderator into four subgroups: pre-school, primary school, secondary school, and university. We divided the control treatment moderator into two categories: studies conducted using traditional methods and studies using media tools. It was important for us to understand the impact of the teacher’s role in the use of AI technology as a tool in vocabulary teaching. In this context, we coded studies where the teacher was at the centre of the teaching process and AI applications were used only as a tool under the heading ‘teacher-led’. We coded studies where AI applications were central to vocabulary teaching and the teacher was only in a supporting role under the heading ‘assistant’. We coded studies where the teaching process was carried out entirely through AI and the teacher was not involved in the process under the heading ‘no teacher’. In the study, we divided the intervention duration heading into three sub-groups: short term, medium term, and long term. Short term covers vocabulary teaching interventions lasting up to 1 week; medium term covers vocabulary teaching interventions lasting up to 1 month; and long term covers interventions lasting longer than 1 month. We divided the intervention setting moderator into two headings: in-school and out-of-school. A significant portion of the studies were conducted in a school setting. The out-of-school heading covers home settings and online interventions.

### 3.4 Quality assessment and risk of bias

To evaluate the methodological quality of the included studies, an adapted risk-of-bias assessment was conducted based on the principles of ROBINS-I. The evaluation process considered key methodological aspects, including the appropriateness of the research design, the presence of a control group, baseline equivalence between groups, clarity of the intervention procedures, the quality of measurement instruments, and the adequacy of outcome data reporting. The assessment indicated that the included studies generally exhibited low to moderate levels of methodological risk. This suggests that the findings of the meta-analysis are not based on a weak evidence base; however, methodological variations across studies should be taken into account when interpreting the results. Nevertheless, small sample sizes, short intervention durations, and limited or insufficient reporting of randomization procedures were identified as the most common methodological limitations. These limitations necessitate a cautious interpretation of the effect sizes and restrict the generalizability of the findings.

### 3.5 Conducting the meta-analysis

In this study, we opted for Hedges’ *g* to determine the effect of AI-supported teaching applications on vocabulary teaching. Indeed, Cohen’s d value may yield biased results in studies conducted with small samples. Hedges’ *g*, on the other hand, expresses the difference between the means of two groups in terms of the overall standard deviation and is more suitable for samples smaller than 20 (Cooper, 2010, pp. 163–168).

Effect sizes were calculated based on the available statistical data reported in each study. When means and standard deviations were reported, Hedges’ *g* was calculated directly using standardized mean differences between experimental and control groups. When studies reported other statistical values (e.g., *t*-values, *F*-values, or *p*-values), these were converted into Hedges’ *g* using standard meta-analytic procedures (Borenstein et al., 2009). All calculations were performed using Comprehensive Meta-Analysis (CMA) software, which applies appropriate formulas depending on the input data.

Additionally, we used the overall effect size and heterogeneity test in the study. *Q* was significant and *I*^2^ exceeded 75. Therefore, we preferred the random effects model and used moderator analyses (Borenstein et al., 2009). We obtained 23 effect sizes for a total of 18 articles. In cases where a single study reported multiple effect sizes, these were included in the meta-analysis based on independent comparisons. In the present study, control treatment was examined as a moderator variable. Therefore, when AI-supported experimental groups were compared with multiple control groups employing different instructional approaches, each comparison was coded as a separate effect size. This approach was adopted to allow a more fine-grained examination of the relative effectiveness of AI-supported interventions across different instructional conditions. However, it is acknowledged that including multiple effect sizes from the same study may introduce a degree of dependency among effect sizes. Although the inclusion of multiple comparisons is considered a common practice in meta-analytic research when they represent distinct comparisons (Borenstein et al., 2009), this potential limitation was taken into account, and the findings were interpreted with caution. Finally, we conducted moderator analyses to determine whether the overall effect size of AI interventions on vocabulary teaching showed significant differences across the moderators of ‘language type, learner educational level, teacher involvement level, control treatment, intervention duration, and intervention setting’.

## 4 Results

**Figure 2:**
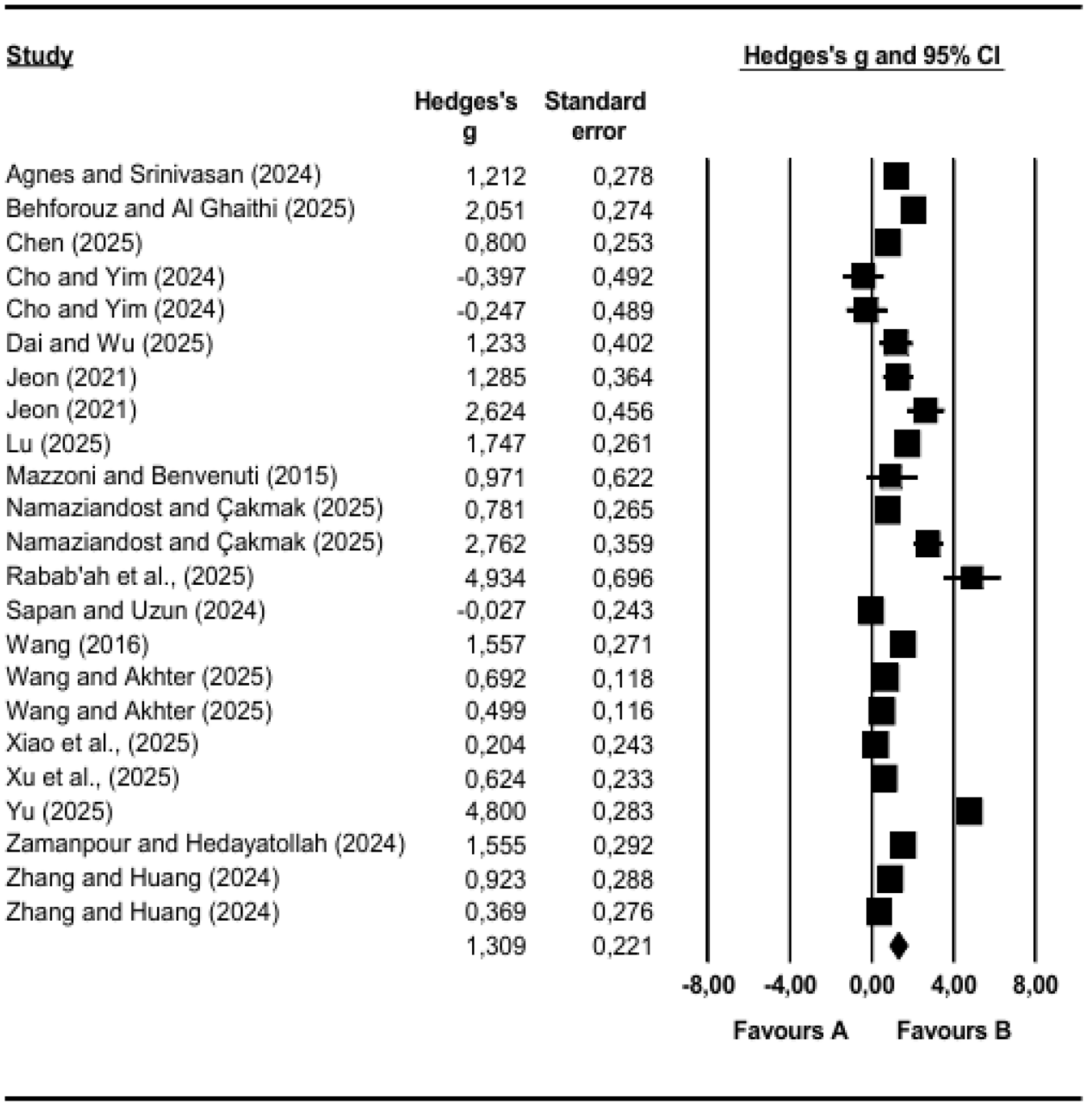
Forest plot of meta-analysis results.

In this meta-analysis study, we first determined the overall effect size of AI-supported interventions on vocabulary learning and the results of the heterogeneity test. The pooled effect size estimate for the use of AI-supported interventions in vocabulary learning success is 1.309 [95% CI (0.876, 1.742), *p* < 0.05]. Within this framework, we found that AI applications have a significant and positive effect on students’ vocabulary learning performance. Our analysis determined that the *I*^2^ value was 93.868%. This result indicates a high level of heterogeneity among the studies included in the meta-analysis. In this context, we can say that the effect of AI-supported interventions may vary depending on the characteristics of the relevant study. We present the overall effect size and heterogeneity test results obtained in the meta-analysis in Table 1 and the forest plot of the effect sizes in Figure 2.

**Table 1:** Overall effect sizes and the heterogeneity test results.

Model	Effect size					Test of heterogeneity			
	*N*	*k*	*g*	SE	95% CI	*Q*	*df*	*p*	*I* ^2^
Random	1823	23	1.309	0.221	[0.876, 1.742]	358.795	22	0.000	93.868

*N*, number of participants; *k*, number of independent comparisons; SE, standard error; CI, confidence interval; *df*, degrees of freedom. ^*^p < 0.05.

The overall effect size (*g* = 1.309) indicates a very large positive effect of AI-supported interventions on vocabulary learning. However, the heterogeneity level was extremely high (*I*^2^ = 93.868%), suggesting substantial variability across studies. This high heterogeneity indicates that the observed effects vary considerably depending on contextual and methodological differences, and therefore the overall effect size should be interpreted with caution.

As shown in Figure 2, the majority of effect sizes favor AI-supported interventions, with most confidence intervals located on the positive side. However, the wide dispersion of effect sizes indicates considerable variability across studies, which is consistent with the high heterogeneity observed.

### 4.1 Sensitivity analysis

A leave-one-out sensitivity analysis was conducted to assess the robustness of the overall effect size. The results showed that the effect size ranged between 1.116 and 1.378 when individual effect sizes were removed. This range indicates that the overall effect size remained generally stable, although some studies had a relatively greater influence on the pooled estimate. In particular, the removal of certain studies [e.g., Yu (2025) and Rabab'ah et al. (2025)] led to more noticeable decreases in the overall effect size, suggesting that these studies contributed more strongly to the pooled estimate. However, none of these changes altered the direction or the overall interpretation of the findings. Additional checks on studies contributing multiple effect sizes yielded similar patterns, further supporting the robustness of the results. Overall, these findings suggest that the observed large effect size is relatively robust, although it should be interpreted with caution in light of potential small-study effects and methodological heterogeneity.

### 4.2 Moderator analysis

As a result of our analysis, owing to the high heterogeneity ratio, we conducted moderator analyses to determine whether the moderator variables could explain the variance detected in the predictions. We present the results in Table 2.

**Table 2:** Moderator analysis.

Moderator variable level	*k*	*g*	SE	95% CI		*Q*	*df*	*p*
				Lower	Upper			
Language type						18.091	1	0.000
1. L1	2	−0.321	0.347	−1.002	0.359			
2. L2	21	1.447^*^	0.229	0.998	1.896			
Learner educational level						11.368	3	0.010
1. Pre-school	3	0.029	0.395	−0.745	0.804			
2. Primary school	2	1.927^*^	0.669	0.615	3.238			
3. Secondary school	4	1.358^*^	0.636	0.112	2.604			
4. University	13	1.548^*^	0.291	0.979	2.118			
Control treatment						4.414	1	0.036
1. Multimedia	9	0.832^*^	0.145	0.548	1.117			
2. Traditional	14	1.633^*^	0.352	0.943	2.323			
Teacher involvement level						2.499	2	0.287
1. Teacher-led	5	1.449^*^	0.667	0.142	2.755			
2. Assistant	13	1.446^*^	0.319	0.821	2.071			
3. No teachers	5	0.855^*^	0.855	0.386	1.324			
Intervention duration						1.245	2	0.536
1. Long-term	8	1.623^*^	0.459	0.724	2.523			
2. Medium-term	7	1.159^*^	0.356	0.460	1.857			
3. Short-term	7	1.036^*^	0.258	0.530	1.543			
Intervention setting						3.915	1	0.048
1. In-school	15	1.606^*^	0.294	1.030	2.182			
2. Out-of-school	4	0.758^*^	0.312	0.146	1.369			

^*^p < 0.05.

The moderator analysis revealed that several variables significantly influenced the effectiveness of AI-supported vocabulary learning. Specifically, language type showed a significant difference, with L2 contexts demonstrating substantially larger effect sizes than L1 contexts. Similarly, educational level was found to be a significant moderator, with stronger effects observed at primary, secondary, and university levels compared to pre-school settings. In terms of control treatment, AI interventions showed stronger effects when compared to traditional instruction than to multimedia-supported instruction, suggesting that the relative advantage of AI may decrease when compared to already enriched learning environments. Additionally, intervention setting was found to be a significant moderator, with in-school implementations yielding stronger effects than out-of-school contexts. In contrast, teacher involvement level and intervention duration did not produce statistically significant differences, indicating that AI-supported learning may be effective across different instructional conditions. We used six moderator variables to determine the potential effects of AI-supported interventions on students’ vocabulary learning performance. The findings regarding these moderators are as follows:

#### 4.2.1 Language type

The language type variable comprises two subgroups: L1 (*k* = 21) and L2 (*k* = 2). According to the analysis results, the effect of AI-supported interventions is larger in the L2 group [*g* = 1.447, 95% CI (0.998, 1.896)]. In contrast, the effect size for the L1 group is relatively smaller [*g* = −0.321, 95% CI (−1.002, 0.359)]. Furthermore, the effect of AI interventions in the L1 context is not significant. The difference between the two groups is significant, *Q*(1) = 18.091, *p* = 0.000. Therefore, it has been concluded that the use of AI-supported teaching applications is more effective in vocabulary teaching in the L2 context. However, the very low number of studies representing the L1 level significantly weakens the reliability and generalisability of the result. Therefore, caution should be exercised when interpreting the results.

#### 4.2.2 Learner educational level

We divided the learner educational level variable into four subgroups. These groups are: pre-school (*k* = 3), primary school (*k* = 2), secondary school (*k* = 4) and university (*k* = 14). We determined that AI-supported interventions had a statistically significant and positive effect at all educational levels except pre-school [*g* = 0.029, 95% CI (−0.745, 0.804)]. The difference between these groups is statistically significant, *Q*(3) = 11.368, *p* = 0.010. The other educational levels are ranked as follows: primary school [*g* = 1.927, 95% CI (0.615, 3.238)], university [*g* = 1.548, 95% CI (0.979, 2.118)], and secondary school [*g* = 1.358, 95% CI (0.112, 2.604)]. Based on the results, we can say that AI-supported interventions have a positive and significant effect on vocabulary teaching in all educational levels except pre-school.

#### 4.2.3 Control treatment

We divided the control treatment moderator into two groups: multimedia (*k* = 9) and traditional (*k* = 14). Experimental procedures supported by AI applications showed a relatively larger effect compared to traditional teaching [*g* = 1.633, 95% CI (0.943, 2.323)]. However, when compared to multimedia-supported teaching, the effect size was considerably high but relatively smaller [*g* = 0.802, 95% CI (0.548, 1.117)]. Furthermore, the difference between the two groups is statistically significant, *Q*(1) = 4.414, *p* = 0.036. In this context, it can be said that AI-supported interventions produce similarly positive results when compared to different teaching processes. Moreover, when compared to traditional interventions, the results are more positive.

#### 4.2.4 Teacher involvement level

We divided the teacher involvement level variable into three subgroups: teacher-led (*k* = 5), assistant (*k* = 13), and no-teachers (*k* = 5). The analysis results showed that all three groups had a significantly positive and large effect size. The effect sizes of the groups are as follows: teacher-led [*g* = 1.449, 95% CI (0.142, 2.755)], assistant [*g* = 1.446, 95% CI (0.821, 2.071)], and no teachers [*g* = 0. 855, 95% CI (0.386, 1.324)]. Furthermore, we determined that there was no statistically significant difference between the groups, *Q*(2) = 2.499, *p* = 0.287. This result indicates that the role of the teacher does not make a significant difference when vocabulary teaching is carried out with AI-supported applications. However, it can be said that students’ vocabulary learning performance increases when the teacher is central to the process or in a supporting position.

#### 4.2.5 Intervention duration

We divided the intervention duration variable into three subgroups: long term (*k* = 8), medium term (*k* = 7), and short term (*k* = 7). Considering the effect sizes, they were, respectively, long term [*g* = 1.623, 95% CI (0.724, 2.523)], medium term [*g* = 1.159, 95% CI (0.460, 1.857)], and short term [*g* = 1.036, 95% CI (0.530, 1.543)]. In this context, it is seen that the increase in intervention duration positively reflects the effect size. However, the difference between the groups is not statistically significant, *Q*(2) = 1.245, *p* = 0.536.

#### 4.2.6 Intervention setting

We examined the intervention setting variable in two groups: in-school (*k* = 15) and out-of-school (*k* = 4). The results show that AI interventions in the school setting [*g* = 1.606, 95% CI (1.030, 2.182)] had a greater effect on vocabulary learning success compared to those in the out-of-school setting [*g* = 0.758, 95% CI (0.146, 1.369)]. The results of our comparison between different intervention settings are statistically significant, *Q*(1) = 3.915, *p* = 0.048. Therefore, it can be said that AI-supported applications in the school setting can improve learners’ vocabulary learning performance more than those in non-school settings.

### 4.3 Publication bias

**Figure 3:**
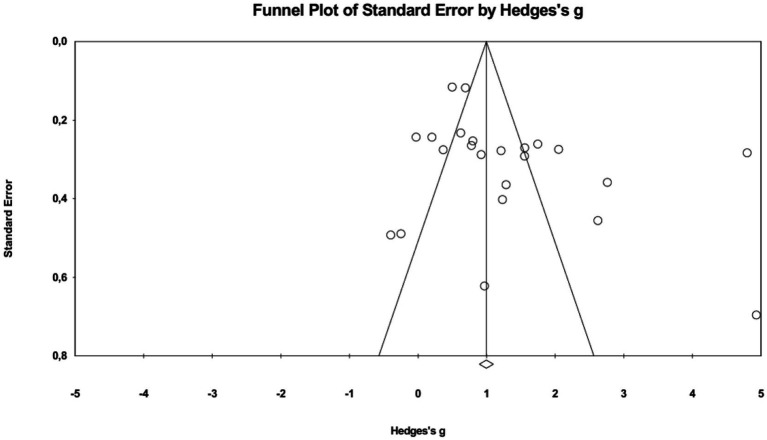
Funnel plot.

In this meta-analysis, we used funnel plots, Egger’s regression test, and Classic fail-safe N to determine publication bias and its effect on the results obtained. If the majority of studies examined in a meta-analysis are statistically significant, publication bias may be present in this analysis (Borenstein et al., 2009). Publication bias indicates the possibility that a selected group of studies published on a subject may not represent all studies (Rothstein et al., 2005/2006). The results and interpretations of our examination in this study are as follows (Figure 3).

Figure 3 shows a relatively symmetrical distribution of studies around the pooled effect size. This visual pattern suggests that there is no strong evidence of substantial publication bias. However, the Egger regression test was not statistically significant but was close to the conventional threshold (*p* = 0.070), indicating that the possibility of small-study effects or mild publication bias cannot be completely ruled out. In addition, the Classic fail-safe *N* result showed that 2,488 additional null-effect studies would be needed to reduce the overall effect to non-significance. Therefore, although the overall findings appear relatively robust, the publication bias results should be interpreted with caution.

## 5 Discussion

The findings revealed that AI-supported interventions appear to have a positive effect on students’ vocabulary learning performance; however, this effect should be interpreted with caution. The overall effect size (*g* = 1.309) indicates that AI-supported interventions have a positive effect on vocabulary learning. However, the high level of heterogeneity (*I*^2^ = 93.868%) indicates substantial variability across studies, suggesting that the observed effect size may not be uniform across different contexts. These results provide concrete evidence of the pedagogical potential of AI interventions in vocabulary teaching. The personalized learning experiences and adaptive content delivery offered by AI systems are thought to play a decisive role in this increase in vocabulary learning performance. Therefore, the results of the current study are consistent with the results of recent studies in the field. Sripada et al. (2025) and Ma et al. (2025) have shown that content difficulty dynamically adjusted according to student performance and personalized systems significantly increase vocabulary acquisition. The instant feedback and interaction opportunities provided by AI tools also facilitate the learning process by adjusting students’ cognitive load (Sweller, 1988; Torres and Kahveci, 2025). Multimodal content (visual, auditory, textual) and associated AI strategies, in line with the principles of the Cognitive Multimedia Learning Theory, increase vocabulary recall rates and positively influence retention (Yu, 2025). Furthermore, AI technologies create a better learning environment by increasing learners’ participation and motivation in the process while reducing foreign language anxiety (Ekizer, 2025; Godwin-Jones, 2021). These cognitive and affective supports may explain the findings in the current study that AI-supported interventions significantly improve students’ performance in vocabulary learning.

As a result of our analysis, we determined that the level of heterogeneity (*I*^2^ = 93.868) was high. In this context, we can say that there was a high level of heterogeneity among the studies included in the meta-analysis. Therefore, depending on the conditions of the different teaching processes carried out with AI intervention, different levels of effects may emerge in vocabulary teaching processes. Although a random-effects model was employed and moderator analyses were conducted to explain this variability, the remaining unexplained heterogeneity suggests that additional factors not included in the present analysis may influence the effectiveness of AI-supported vocabulary learning. These factors may include differences in AI system design, degree of personalization, instructional context, learner characteristics, and implementation fidelity. Therefore, the pooled effect size reported in this study should be interpreted as an average tendency rather than a precise estimate of a uniform effect. The findings highlight that the effectiveness of AI-supported interventions is highly context-dependent, and caution should be exercised when generalizing the results across different educational settings. Indeed, similar effects at different levels have been reported in the literature (Ekizer, 2025, *I*^2^ = 92.66; Torres and Kahveci, 2025, *I*^2^ = 88). The effect sizes of AI interventions in the literature vary widely. While some studies found very large effects (Lee et al., 2025, *g* = 3.04; Ouyang et al., 2024, *g* = 3.57; Yu, 2025), others found no significant difference (Liu et al., 2025; ElEbyary et al., 2024, *g* = −0.02 and *g* = −0.15), or limited effects have been reported (Burston, 2015). Furthermore, it has been determined that AI is effective in receptive knowledge but more limited in productive use (Alisoy and Sadigzade, 2025; Li and Hafner, 2022) and that positive effects may diminish over the long term (Temiz and Kafadar, 2025). These findings may explain the high heterogeneity observed in this study and suggest that the effect of AI may vary depending on contextual factors. Accordingly, although the overall findings indicate a positive effect of AI-supported interventions, these results should be interpreted with caution. The methodological limitations of the included studies introduce a degree of uncertainty regarding the consistency and generalizability of the findings. Factors such as variation in study designs, differences in intervention characteristics, and limited sample sizes across some subgroups may have influenced the results. Therefore, the effectiveness of AI interventions may vary depending on specific educational contexts rather than representing a universally strong effect.

According to the analysis results, the effect of AI-supported interventions is greater in the L2 group. In contrast, the effect size of the L1 group is relatively smaller and not significant. The difference between the two groups is significant. It is seen that the vast majority of L2 studies consist of English teaching studies. Therefore, it has been concluded that the use of AI-supported teaching applications is more effective in vocabulary teaching in the L2 and English contexts. However, the very low number of studies representing the L1 level significantly weakens the reliability and generalizability of the results. Research in the field reports that studies conducted in the context of AI have focused quite frequently on English teaching and have generally yielded positive learning outcomes (Ekizer, 2025; Torres and Kahveci, 2025). In the context of the difference between L1 and L2, our findings are consistent with the meta-analysis conducted by Şimşek and Şimşek (2025), which found that “technology-supported vocabulary teaching creates a much higher effect size in the L2 context (*g* = 1.256) compared to L1 (*g* = 0.590).” Furthermore, Qiao et al. (2025) and Zhou and Zhou (2026) emphasize that the majority of studies in the literature focus on English language learning. Therefore, more studies covering languages other than English are needed to fully assess the impact of AI tools on a large scale and across different languages (Torres and Kahveci, 2025; Zhou and Zhou, 2026).

We also examined the educational level of the learners as a moderator. We determined that AI-supported interventions had a meaningful and positive effect at all educational levels except preschool. The difference between these groups is statistically significant. The other educational levels are ranked in order as primary school, university, and secondary school. Based on the results, it can be said that AI-supported interventions have a positive and significant effect in the context of vocabulary teaching at all educational levels except preschool. This finding is consistent with the results obtained by Xu and Wang (2024) and Torres and Kahveci (2025), particularly in terms of the high effect size at the primary school level. However, the lack of a significant effect in the preschool group contradicts the meta-analysis findings by Şimşek and Şimşek (2025), which determined that technology-supported vocabulary teaching (*g* = 0.729) is effective in the preschool period. In contrast, some meta-analyses at the university level (Wu, 2024; Yu and Trainin, 2022; Zhou and Zhou, 2026) have argued that students at this level benefit more from technology due to their advanced self-regulation and motivation. From this perspective, AI-supported interventions appear to be more effective in higher education than in K-12, where effects vary across different levels.

According to the analysis results, experimental procedures supported by AI applications have shown a relatively greater effect compared to traditional teaching. This finding is consistent with the results of meta-analyses conducted by Ekizer (2025) and Torres and Kahveci (2025). However, although the effect size is high when compared to multimedia-supported teaching, it is relatively lower than that of traditional teaching. Furthermore, in both comparisons, the difference between the groups is statistically significant. In the literature, Yu (2025) and Kanellopoulou et al. (2019) emphasize that multimodal approaches, in line with the Cognitive Multimedia Learning Theory (CMLT), increase learning outcomes by presenting visual and auditory elements together. In this context, it can be said that AI-supported interventions yield similarly positive results when compared to different teaching processes. Moreover, these results appear more positive when compared to traditional interventions. The main reason for this difference is the personalization and adaptability features offered by AI, which go beyond standard multimedia tools (Ma et al., 2025; Simonnet et al., 2025).

We examined the teacher involvement level variable in three subgroups: teacher-led, assistant, and no teachers. The analysis results showed that all three groups had a significantly positive and large effect size. We also determined that there was no statistically significant difference between the groups. This result indicates that the teacher’s role does not make a significant difference when word teaching is carried out with AI-supported applications. However, it can be said that students’ word learning performance increases when the teacher is central to the process or in a supporting role. This finding is consistent with studies demonstrating that AI tools can also function effectively in autonomous learning environments (Kakumanu, 2025; Lin and Lin, 2019). However, qualitative data in the literature emphasizes that the teacher’s role is critical in terms of the depth of learning, even if there is no statistical difference. Indeed, Alisoy and Sadigzade (2025) state that mobile applications are superior in terms of memorization, but teacher-led methods are much more effective in terms of productive language use of words. Similarly, Wiboolyasarin et al. (2025) and Torres and Kahveci (2025) state that the impact of AI reaches its highest level when teachers guide the process. Therefore, the absence of intergroup differences in the current study can be interpreted not as AI replacing the teacher, but as the teacher’s role needing to shift from “knowledge transmitter” to “process facilitator” (Moslemi Nezhad Arani, 2025).

The intervention duration variable is divided into three subgroups: long-term, medium-term, and short-term. Considering the effect sizes, the interventions are ranked in order of long-term, medium-term, and short-term; in this context, it is observed that an increase in duration has a positive effect on the effect size. However, the difference between the groups was not found to be statistically significant. This finding is consistent with the results of the meta-analysis conducted by Zhou and Zhou (2026) in terms of emphasizing the effectiveness of long-term applications. However, there is no consensus on this issue in the literature. For example, Wu, 2024 and Xu and Wang (2024) stated that medium-term interventions provide the highest impact, while success may decline in very long-term applications due to a decrease in student interest (learner fatigue) and the loss of the novelty effect. The fact that the difference between the groups was insignificant in the current study suggests that the nature of the intervention, rather than its duration, is the determining factor.

The analysis conducted in terms of the learning environment shows that AI interventions implemented in the school environment have a greater and more significant effect on vocabulary teaching success compared to out-of-school environments. This result differs from the findings of researchers such as Lin and Lin (2019), who found that out-of-school and informal environments that support autonomous (independent) learning have a higher impact. However, the findings of the current study are consistent with studies that highlight the importance of teacher guidance. Simonnet et al. (2025) note that technology has the highest impact in academic contexts and with teacher guidance, while Alisoy and Sadigzade (2025) emphasize that traditional classroom environments play a critical role in the development of productive language skills. Similarly, Temiz and Kafadar (2025) state that classroom practices at the K-12 level facilitate students’ adaptation to technology and process tracking. Therefore, it can be said that AI-supported applications in the school environment can increase students’ vocabulary learning success more than out-of-school environments thanks to the social interaction and teaching support they offer.

## 6 Conclusion

The results of this study indicate that AI-supported interventions can improve students’ vocabulary learning performance. Furthermore, the overall effect size [*g* = 1.309, 95% CI (0.876, 1.742), *p* < 0.05] demonstrates that the effect of AI use in the context of vocabulary teaching is positive and large. The level of heterogeneity identified in the study (*I*^2^ = 93.868%) reveals that the effect of AI can vary across different studies. To understand the source of this variation, we conducted a moderator analysis based on the variables “language type, learner educational level, teacher involvement level, control treatment, intervention duration, and intervention setting.” The results were significant for the moderators language type, learner educational level, control treatment, and intervention setting. According to the findings, AI interventions yielded more efficient results in the L2 context. In this context, it appears that AI can offer advantages in the process of learning a new language, particularly in the context of vocabulary teaching. In addition, students’ vocabulary learning performance had a significant effect at all educational levels except preschool. Therefore, it can be said that AI-supported interventions produced more effective results starting from primary school. One of the important findings of the study is that AI interventions had a significant effect size when compared to traditional methods, but this effect decreased when compared to media-supported interventions. We concluded that studies conducted in an additional school environment could be preferred for increasing students’ vocabulary learning performance. The meta-analysis showed that the results were not significant in terms of teacher involvement level and intervention duration moderators. However, when teachers were active in the classroom, students’ AI-supported vocabulary learning performance increased. In addition, better learning performance was recorded in groups with longer intervention periods. When the results are evaluated overall, it can be said that AI interventions have a significant and positive effect on students’ vocabulary learning performance.

### 6.1 Limitations and recommendation

This study has several methodological limitations that should be considered when interpreting the findings. First, the meta-analysis is based on a relatively small number of studies (18 studies and 23 effect sizes), which may limit the generalizability of the results. Second, the very high level of heterogeneity (*I*^2^ = 93.868%) indicates substantial variability across studies that is not fully explained by the selected moderator variables. This suggests that other unexamined factors may influence the effectiveness of AI-supported vocabulary learning. Third, although the literature search was limited to three major databases (Web of Science Core Collection, Scopus, and ERIC), this selection was intentional to ensure the inclusion of high-quality, peer-reviewed studies. These databases are widely recognized for their rigorous indexing criteria and are commonly used in meta-analytic research. However, it is acknowledged that studies indexed in other sources such as Google Scholar, IEEE Xplore, ACM Digital Library, or other specialized repositories may have been excluded. Therefore, potential publication bias and database bias should be considered when interpreting the findings. Furthermore, this study was limited to the years 2015–2025, and future reviews may yield different findings by including broader time frames. In addition, the studies reviewed focused primarily on English language learning contexts and L2 populations, which may limit the generalizability of the findings to other linguistic settings. Therefore, the results of this meta-analysis should be interpreted with caution.

The results of this meta-analysis may encourage further research to provide a deeper understanding of the potential of utilizing AI applications to enhance students’ vocabulary learning performance. Future research may consider broader time frames. The studies we reviewed focused primarily on English language teaching and L2. In this context, it is thought that the frequency of AI-supported studies targeting different languages should be increased. This would allow for more generalizable findings. A significant portion of the studies included in the meta-analysis were conducted with university students. Therefore, it may be advisable to focus on studies with younger age groups. Another prominent finding of the study was that AI-supported interventions yielded much more positive results, especially when compared to traditional interventions. Based on these results, we believe that educators can enrich their lesson plans, which are primarily designed using traditional methods, by integrating AI technology and achieve more positive outcomes. Another result that emerged from the study was related to the role of the teacher in the AI-supported teaching process. Although the difference between the groups was not significant, the results were more positive in AI-supported vocabulary teaching processes where teachers were in an active position. Therefore, it may be more beneficial for teachers to create AI-supported content where they are central or in a supporting position when preparing lesson content. In addition, the results suggest that longer-term interventions may be associated with higher performance. Considering that the school environment also significantly increases vocabulary learning performance, intervention plans that are teacher-centered, implemented in the school environment, and carried out over a long period of time may be recommended to increase learning success.

## Data Availability

The data presented in this study are available on reasonable request from the corresponding author.

## Appendix A

WOS: TI=((“artificial intelligence” OR “AI” OR “chatbot” OR “ITS” OR “ALS” OR “robot”) AND (“vocabulary” OR “language learning” OR “word”) AND (“learning achievement” OR “learning performance” OR “academic outcome” OR “acquisition”) NOT (“meta-analysis” OR “systematic review” OR “literature review”)) OR AB = ((“artificial intelligence” OR “AI” OR “chatbot” OR “ITS” OR “ALS” OR “robot”) AND (“vocabulary” OR “language learning” OR “word”) AND (“learning achievement” OR “learning performance” OR “academic outcome” OR “acquisition”) NOT (“meta-analysis” OR “systematic review” OR “literature review”)).

Scopus: Search within (Article title, Abstract, Keywords).

(“artificial intelligence” OR “AI” OR “chatbot” OR “ITS” OR “ALS” OR “robot”) AND (“vocabulary” OR “language learning” OR “word”) AND (“learning achievement” OR “learning performance” OR “academic outcome” OR “acquisition”) AND NOT (“meta-analysis” OR “systematic review” OR “literature review”)

ERIC: TI=((“artificial intelligence” OR “AI” OR “chatbot” OR “ITS” OR “ALS” OR “robot”) AND (“vocabulary” OR “language learning” OR “word”) AND (“learning achievement” OR “learning performance” OR “academic outcome” OR “acquisition”)) OR AB = ((“artificial intelligence” OR “AI” OR “chatbot” OR “ITS” OR “ALS” OR “robot”) AND (“vocabulary” OR “language learning” OR “word”) AND (“learning achievement” OR “learning performance” OR “academic outcome” OR “acquisition”) NOT (“meta-analysis” OR “systematic review” OR “literature review”)).
